# Supplementary material for: Detailed molecular and epigenetic characterization of the pig IPEC-J2 and chicken SL-29 cell lines
Source: iScience. 2023 Feb 20;26(3):106252. doi: 10.1016/j.isci.2023.106252 (PMC10018572; doi:10.1016/j.isci.2023.106252)
Supplement: Data S1. Complete homer output for identified motifs in Pig IPECJ-2, related to Table 2 — Homer motif analysis results for histone modifications H3K4me1, H3K4me3, H3K27ac, and enhancer elements of pig IPECJ2 cell line. P-values >1e-10 are possible false positives. Within each folder (e.g. peak_files_CTCF) are the html files showing the identified motifs when using homer (e.g. homerResults.html). [file mmc2.zip › S5/Pig_IPECJ_2/peak_files_enhancer/homerResults/motif6.similar.html]

motif6

## Information for motif6

G
A
C
T
C
A
G
T
G
T
C
A
A
G
T
C
C
G
A
T
A
C
T
G
G
A
T
C
A
T
G
C
C
A
G
T
C
A
T
G
  
Reverse Opposite:  

G
T
A
C
G
C
T
A
T
A
C
G
C
T
A
G
T
G
A
C
C
G
T
A
T
C
A
G
A
C
G
T
G
T
C
A
C
T
G
A
  

|  |  |
| --- | --- |
| p-value: | 1e-56 |
| log p-value: | -1.306e+02 |
| Information Content per bp: | 1.650 |
| Number of Target Sequences with motif | 3218.0 |
| Percentage of Target Sequences with motif | 8.78% |
| Number of Background Sequences with motif | 2421.0 |
| Percentage of Background Sequences with motif | 6.62% |
| Average Position of motif in Targets | 149.8 +/- 83.9bp |
| Average Position of motif in Background | 148.4 +/- 91.5bp |
| Strand Bias (log2 ratio + to - strand density) | -0.0 |
| Multiplicity (# of sites on avg that occur together) | 1.06 |
| Motif File: | file (matrix) reverse opposite |

### Similar de novo motifs found

|  |  |  |  |  |  |  |  |
| --- | --- | --- | --- | --- | --- | --- | --- |
| Rank | Match Score | Redundant Motif | P-value | log P-value | % of Targets | % of Background | Motif file |
| 1 | 0.634 | A T G C G A C T A T C G A G T C A G T C A G T C C A T G T C A G | 1e-48 | -111.265771 | 30.63% | 27.18% | motif file (matrix) |
| 2 | 0.651 | C G T A A T C G A T G C G A C T T A G C C G T A C A T G A T C G T G A C G T C A A C G T A G C T C G T A | 1e-47 | -109.309011 | 0.13% | 0.01% | motif file (matrix) |
| 3 | 0.601 | T G A C A T G C C T A G A T C G A T C G T A G C A G T C A C T G | 1e-47 | -108.341520 | 27.75% | 24.46% | motif file (matrix) |
| 4 | 0.610 | A C T G A G T C A G T C C G A T A C T G G T C A A G T C C G T A | 1e-21 | -49.105011 | 6.85% | 5.66% | motif file (matrix) |
